# Rapid Consumption of Dihydrogen Injected into a Shallow Aquifer by Ecophysiologically Different Microbes

**DOI:** 10.1021/acs.est.3c04340

**Published:** 2023-12-20

**Authors:** Nina S. Keller, Klas Lüders, Götz Hornbruch, Susann Birnstengel, Carsten Vogt, Markus Ebert, René Kallies, Andreas Dahmke, Hans H. Richnow

**Affiliations:** † Department of Isotope Biogeochemistry, Helmholtz Centre for Environmental Research—UFZ, Permoserstr. 15, 04318 Leipzig, Germany; ‡ Department of Applied Geosciences - Aquatic Geochemistry and Hydrogeology, Institute for Geosciences, Competence Centre for Geoenergy (KGE), 24118 Kiel, Germany; § Department of Monitoring & Exploration Technologies, Helmholtz Centre for Environmental Research—UFZ, Permoserstr. 15, 04318 Leipzig, Germany; ∥ Department of Environmental Microbiology, Helmholtz Centre for Environmental Research—UFZ, Permoserstr. 15, 04318 Leipzig, Germany; ⊥ Isodetect GmbH, Deutscher Platz 5b, 04103 Leipzig, Germany

**Keywords:** H_2_ economy, hydrogenotrophy, field experiment, H_2_ gas release, shallow groundwater, microbial community, diversity

## Abstract

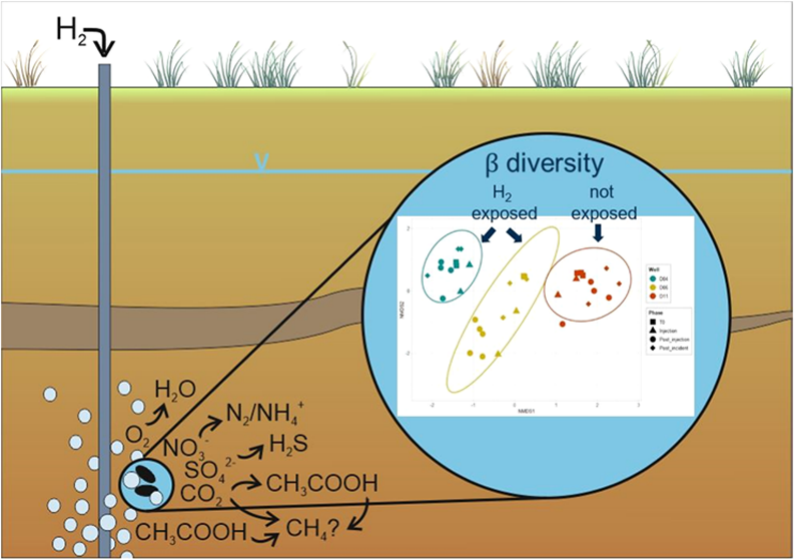

The envisaged future dihydrogen (H_2_) economy requires a H_2_ gas grid as well as large deep underground stores. However, the consequences of an unintended spread of H_2_ through leaky pipes, wells, or subterranean gas migrations on groundwater resources and their ecosystems are poorly understood. Therefore, we emulated a short-term leakage incident by injecting gaseous H_2_ into a shallow aquifer at the TestUM test site and monitored the subsequent biogeochemical processes in the groundwater system. At elevated H_2_ concentrations, an increase in acetate concentrations and a decrease in microbial α-diversity with a concomitant change in microbial β-diversity were observed. Additionally, microbial H_2_ oxidation was indicated by temporally higher abundances of taxa known for aerobic or anaerobic H_2_ oxidation. After H_2_ concentrations diminished below the detection limit, α- and β-diversity approached baseline values. In summary, the emulated H_2_ leakage resulted in a temporally limited change of the groundwater microbiome and associated geochemical conditions due to the intermediate growth of H_2_ consumers. The results confirm the general assumption that H_2_, being an excellent energy and electron source for many microorganisms, is quickly microbiologically consumed in the environment after a leakage.

## Abstract 2

For the first time, the response of a shallow groundwater microbiome to temporarily elevated levels of gaseous dihydrogen was assessed in a field experiment. Significant but temporally limited effects on microbial diversity and community composition were observed.

## Introduction

The dihydrogen (H_2_) economy is an aspired future in which H_2_ is used as an energy carrier and raw material and therefore requires both a gas network for transport and storage options.^1,2^ Subsurface storage in caverns, mines, or natural porous rock formations (like depleted gas or oil fields or deep aquifers)^3^ enables an economically viable long-term storage of large quantities of H_2_ in the deep subsurface.^2,4−7^ The risks of geological H_2_ storage technologies have not yet been fully investigated although leakages of stored gas through geological fractures, for instance, have been reported regularly at underground gas storage (UGS) sites where gas mixtures containing up to 95% H_2_ can be stored.^8,9^

Biogenic H_2_ is mainly produced by dark fermentation of organic substrates in anoxic soil habitats; however, concentrations are usually low due to the immediate consumption of generated H_2_ by anaerobic hydrogenotrophs already in the anoxic areas or aerobic hydrogenotrophs at oxic–anoxic interfaces.^8,10,11^ Both bacteria and archaea belong to these hydrogenotrophs, which use H_2_ as an electron donor to generate energy. Generally, hydrogenase, a metal-containing enzyme that exists in different forms, catalyzes the conversion of H_2_ into protons and electrons (as well as the reverse reaction) in all hydrogenotrophic microorganisms.^12^ Relevant electron acceptors of hydrogenotrophs with decreasing energy yield are oxygen (O_2_) as well as nitrate (NO_3_^–^), nitrite (NO_2_^–^), ferric iron (Fe^3+^), sulfate (SO_4_^2–^), and carbon dioxide/bicarbonate (CO_2_/HCO_3_^–^).^13^ The latter refers to hydrogenotrophic methanogenesis and acetogenesis via H_2_ oxidation and CO_2_/HCO_3_^–^ reduction. During anaerobic digestion of organic matter (OM), H_2_ is an important electron carrier of reducing equivalents which are transmitted directly between cells of H_2_ producers and consumers (termed interspecies H_2_ transfer), facilitating syntrophic interactions.^10,14^ Since hydrogenotrophy is widely distributed within different taxonomic and ecophysiological groups, hydrogenotrophic prokaryotes can be common in aquifers. Many hydrogenotrophs are able to switch their energy metabolism and grow on other (organic) substrates as well to ensure survival when H_2_ is not available.^10,11^

The most relevant microbial H_2_-consuming processes expected at H_2_-UGS sites are methanogenesis, sulfate reduction, as well as acetogenesis, which can possibly occur simultaneously (in different microenvironments) when H_2_ is stored in high concentrations.^9,15,16^ Gas migrations from deep reservoirs or leakages in gas pipelines into the shallow subsurface could potentially affect shallow groundwater resources by H_2_-oxidation processes and stimulate microbial activity. The production of toxic and corrosive dihydrogen sulfide (H_2_S) by H_2_-driven microbial sulfate reduction is of particular importance.^8^ Furthermore, the production of methane (CH_4_) may partly foil the climate protective usage of H_2_ because CH_4_ is a strong greenhouse gas. Previous laboratory studies examining the biogeochemical effects of millimolar H_2_ concentrations^15,17,18^ found Fe^3+^ and sulfate reduction as well as acetogenesis as predominant H_2_-consuming processes under anoxic conditions. In a previous in situ study in the Opalinus Clay formation at the rock laboratory Monte Terri, the response of a deep subsurface microbial community to gaseous H_2_ was investigated, demonstrating H_2_ oxidation by different ecophysiological groups.^19^ However, environmental conditions of the Opalinus Clay differ from those normally found in shallower areas, e.g., due to different temperatures, geologic textures, pressures, and salinities.^9^ Thus, it is unknown whether the results of this study are transferable to shallower aquifers. Recently, a short H_2_ leak was simulated in a shallow unconfined aerobic chalk aquifer in France by injecting H_2_-saturated groundwater; notably, microbial consumption of H_2_ was not observed in this field study due to the limited amount of dissolved H_2_ added (9 g in total), a rapid transfer of the dissolved H_2_ plume through the aquifer, and its significant dilution downstream of the injection well.^20^

The aim of this field-scale study was to assess the effects of a gaseous H_2_ leakage from, e.g., a UGS on the groundwater microbiome in a shallow aquifer by a controlled three-day H_2_-release experiment. Stable isotope effects associated with H_2_ injection, H_2_ transport processes, and putative microbial consumption in the course of the field experiment have recently been described.^18^ Here, we determined the time response and mode of microbial H_2_ consumption until recovery from initial biogeochemical conditions. To the best of our knowledge, the effects of the entry of gaseous H_2_ on a groundwater microbiome and the associated redox reactions in a shallow aquifer have not been investigated in situ so far.

## Materials and Methods

### Experimental Site and Setup

The hydrogeological TestUM test site is located near Wittstock/Dosse (Brandenburg, Germany) approximately 100 km from Berlin (Figure S1). Previously, a CO_2_ injection test^21−24^ and a five-day infiltration test of ∼75 °C hot water^25−27^ were conducted in other areas of the test site (Figure S1). The corresponding studies include a detailed description of the location and give an overview of the wider geological conditions. The geological conditions in the area of the gaseous H_2_ injection test as well as the injection methodology are summarized in the Supporting Information (SI) and were also described in our previous study in which we reported on the stable isotope effects associated with the H_2_ injection.^18^

### Groundwater Sampling

Groundwater was sampled as previously described;^18,27^ a detailed description is given in the Supporting Information (SI-1–3). The sampling wells were selected according to a previous study for monitoring isotopic effects,^18^ i.e., groundwater was taken from 11.5, 14.5, or 17.5 m from wells D04, D06, and D11 and subsequently analyzed, respectively. Wells D04 and D06 are located in close vicinity (<5 m) and downstream of the injection spots, whereas well D11 is located around 25 m southeast of the injection, functioning as a H_2_-free control (Figure S1B). The initial states of the hydrogeochemical conditions and the groundwater microbiome were determined 19–20 days before the H_2_ injection (henceforth referred to as T0 sampling). Groundwater samples were taken on days 2 and 3 of the three-day H_2_ injection. Post injection (henceforth referred to as postinjection phase), groundwater was sampled after 1 day (solely from well D06), and after 5–6, 13–14, 19–20, and 26–27 days. When the H_2_ concentrations fell below the detection limit (henceforth referred to as postincident phase), further groundwater samples were collected, specifically, 76–77, 214, and 243–244 days after the injection.

Each sample used for 16S ribosomal ribonucleic acid (rRNA) gene amplicon sequencing consisted of 4 L of groundwater. For total cell counting, 5 mL of groundwater was fixed on the site with 5 mL (vol/vol) of 4% paraformaldehyde (PFA) in a sterilized 50 mL serum bottle that was sealed gastight with sterilized butyl rubber stoppers and aluminum crimp seals as described in detail elsewhere.^26^ All samples for microbiological analyses were taken from 14.5 m well depth and kept at around 4 °C in a fridge on-site (maximum 2 days) and transported cooled to the Helmholtz Centre for Environmental Research in Leipzig (Germany), where they were processed within 3 days.

### Hydrogeochemical Analyses

Methods to determine physicochemical on-site parameters (temperature, pH, Eh, electrical conductivity, and O_2_ concentrations), alkalinity, sulfide (HS^–^), and H_2_ have been described elsewhere.^18^ The main inorganic anions as well as acetate (CH_3_COO^–^) and formate (HCOO^–^) were determined by ion chromatography (IC 881, Metrosep A Supp 5-150/4.0; Deutsche Metrohm GmbH & Co. KG; Germany). The main inorganic cations including dissolved iron and manganese were analyzed with an ICP-OES (Vista AX; Varian Medical Systems, Inc.; CA). For determining the concentrations of nonpurgeable organic carbon (NPOC), a TOC/TN analyzer (multi N/C 2100; Analytik Jena AG; Germany) was used. Chlorinated ethenes and C1–C5 hydrocarbons were measured by headspace gas chromatography equipped with an ECD and FID detector (GC 6890plus, HS7694; Agilent Technologies, Inc.; CA).

To gain the amount of potential electron acceptors in the solid phase, the reactive Fe^3+^ content of 15 sediment samples from the injection horizon of wells U03 and U05 was determined by extraction with 1 M HCl as described in Leventhal and Taylor^28^ and subsequent photometric analyses of the extracts using the ferrozine method,^29^ whereby hydroxylammonium chloride functions as a reductive agent.

### Amplicon Sequencing

The samples for the microbial diversity analyses were processed as previously described.^26^ The V3–V4 hypervariable region of the *16S rRNA* gene was amplified according to Klindworth et al.^30^ using a 2× MyTaq Mix (Bioline; Heidelberg; Germany), whereby the primer pair S-D-Bact-0341-b-S-17–S-D-Bact-0785-a-A-21 was chosen due to its good bacterial diversity coverage.^30^ The libraries for sequencing were prepared as described in the Illumina 16S Metagenomic Sequencing Library Preparation protocol.^31^ The pool with 4 nM libraries was run on an Illumina MiSeq system (Illumina; CA) using v3 600 cycles chemistry.

The paired-end, demultiplexed fastq files were analyzed utilizing Quantitative Insights Into Microbial Ecology 2 (QIIME 2) version 2021.11^32^ and a pipeline that was initially provided by Dr. Denny Popp (University of Leipzig Medical Center; Germany) but modified to analyze this data set. The primer sequences were removed, and untrimmed reads as well as reads <50 base pairs (bp) were discarded using cutadapt version 2.10.^33^ Denoising was done with the QIIME 2 DADA2 plugin; this included trimming (at positions 284 and 202 of the forward and reverse reads, respectively), quality filtering, learning of the error rates, dereplicating, chimera removal, and merging of paired-end reads.^34^ Since the samples were sequenced on two Illumina MiSeq runs, DADA2 was run separately on data obtained from the respective run. The denoised data were merged before taxonomic annotation using the SILVA 138 (99%) data set,^35,36^ which had been trained with the primer pair used to amplify the V3–V4 hypervariable region of the *16S rRNA* gene in advance. Reads classified as chloroplasts as well as mitochondria were removed (reads classified as Eukaryota or unassigned reads were not present in the data set). *16S rRNA* gene sequences were deposited at the European Nucleotide Archive (ENA) under accession number PRJEB56759.

### Total Cell Counting

The methodology was used for total cell counting has been described in Keller et al.^26^ Cells were counted manually using ImageJ 1.53e along with the *cell counter* plugin.

### Figures and Statistical Analyses

Figures and statistical analyses were conducted with RStudio version 2022.02.1.+461.^37^ The detailed procedure including all used functions and packages is described in the SI (SI-1). The Shannon–Wiener indices were used as a proxy for the α-diversity, and to illustrate the β-diversity, nonmetric multidimensional scaling (NMDS) was performed.

## Results and Discussion

### Hydrogeochemical Changes Due to H_2_ Injection

Data of geochemical and physicochemical parameters in groundwater samples from wells D04, D06, and D11 at different depths (11.5, 14.5, and 17.5 m) before and after H_2_ injection are shown in Figures S2–S6. Injection of gaseous H_2_ into the saturated zone of the aquifer is expected to result in the fast dispersion of gas-phase H_2_ through channels in different directions, accompanied by stripping of other gases, displacement of water, and slow dissolution of H_2_ in the aqueous phase.^18^ During H_2_ injection, the aqueous H_2_ concentrations increased to up to 600 μM in well D04 at 14.5 m depth, whereby similar amounts were detected at 17.5 m depth (Figure S2A). The aqueous H_2_ concentrations of well D06 generally rose until 19–20 days post injection when the highest values of around 830 μM were observed at 11.5 and 14.5 m depths (Figure S2E). 76–77 days post injection and afterward (until the last sampling for this study), H_2_ was no longer detectable in any groundwater samples at any depths of wells D04 and D06 (Figure S2A,B), respectively. No H_2_ was detected in the control well D11 throughout the whole monitoring period (Figure S2I).

H_2_ injection went along with low oxygen concentrations (Figure S6C,F) and sharply decreasing redox potentials to −121 mV in well D04 (Figure S2B) and to −249 mV in well D06 (Figure S2F), indicating the rapid development of more reducing redox conditions in both wells. In well D04, the redox potential at 14.5 m depth stayed at negative values in the course of the experiment, whereas in well D06, the redox potential increased to +164 mV 43 days after H_2_ injection and slowly decreased to negative values afterward. Initial reducing redox conditions in wells D04 and D06 upon H_2_ injection were also indicated by increased concentrations of dissolved iron species (Figure S6A,D). In contrast, redox potentials of the control well D11 were always positive, and dissolved iron species were below the detection limit; however, redox potentials sharply decreased 20 days after H_2_ injection from around +400 mV to less than +100 mV within a week, but increased later on to values of above +300 mV, which can probably be attributed to a combination of groundwater displacement and mixing processes induced by gaseous H_2_ injection. The control well D11 might be influenced as well by groundwater being displaced from the injection area by preferential flow in the southeast direction;^18^ this assumption is supported by the appearance of small amounts (<15 nM) of trichloroethene (TCE) at all depths of the control well D11 in the early postinjection phase and later on (Figure S4L), indicating mobilization of TCE-containing groundwater from other aquifer areas. TCE was detected already before H_2_ injection at some depths of wells D04 and D06 (Figure S4D,H). Chlorinated aliphatics were probably introduced into the aquifer during the usage of the site as a military airport in the former Germany Democratic Republic.^21,22^

The concentrations of major cations (Na^+^, Ca^2+^, Mg^2+^) and anions (SO_4_^2–^, Cl^–^) were predominantly stable during and after H_2_ injection (Figures S3 and S5). Notably, NO_3_^–^ was not detectable at all in well D04 and below 40 μM in two depths of well D06 (11.5, 14.5 m) before H_2_ injection; NO_3_^–^ quickly disappeared after H_2_ injection in well D06 and returned after 78 days at 11.5 m depth at similar concentrations as detected before H_2_ injection, but it was detected only sporadically at 14.5 and 17.5 m depths in the postinjection phase (Figure S3E). NO_2_^–^, a typical metabolite of dissimilatory NO_3_^–^ reduction, was not detected in wells D04 and D06 (Figure S3A,F). In the control well D11, NO_3_^–^ concentrations were between 80 μM (17.5 m) and 300 μM (11.5 m) before H_2_ injection; the values leveled off to 130–210 μM NO_3_^–^ during and after H_2_ injection (Figure S3I), indicating mixing processes. At a depth of 17.5 m, some NO_2_^–^ (<30 μM) was detected before and after H_2_ injection (Figure S3J), indicating ongoing microbial NO_3_^–^ reduction.

In our previous study, we observed an equilibrium isotope fractionation of H_2_ in the course of its depletion, which was interpreted to be caused by microbial oxidation due to the enzyme hydrogenase;^18^ this hypothesis is supported by the transient increasing concentrations of acetate and formate in wells D04 and D06 in the postinjection phase (Figure S4A,B,E,F) and the absence of these compounds in the control well D11 (Figure S4I). Formate is a metabolite and acetate is an end product of the microbial conversion of H_2_ and CO_2_ upon homoacetogenesis;^38^ acetate formation from H_2_ and CO_2_ is usually accompanied by a transient accumulation of formate.^39^ CH_4_, indicative of methanogenesis, was detected above background concentrations in the early postinjection phase once in well D04 (Figure S4C), but at the same time also in the control well D11 (Figure S4K). Hence, geochemical data did not indicate a relevant H_2_-driven methanogenesis in this phase. Higher CH_4_ concentrations (up to 27 μM) were sporadically observed in the late postinjection phase in deeper zones of well D06 (Figure S4G) and were not linked to more negative redox conditions (Figure S2F) typical for methanogenesis; we cannot rule out that CH_4_ was formed in other areas of the site and transported to well D06.

In summary, the hydrogeochemical and physicochemical data show that the aquifer was saturated with H_2_ close to the injection well, accompanied by slightly changing hydrogeochemical conditions. A part of these data and previously reported stable isotope data indicated that H_2_ was microbiologically consumed in the aquifer and that at least a part of the H_2_ was oxidized by homoacetogens. To obtain a more detailed view of the microbiological processes connected to the injection of H_2_, we frequently determined the composition of the microbial communities of the H_2_-exposed wells D04 and D06 and the control well D11 before, during, and after H_2_ injection.

### Microbial Diversity Changes Due to H_2_ Injection

Generally, due to the multidirectional spatial sampling (especially in 2 in. wells) and the volume of 4 L, each sample could capture a mixture of different microenvironments in the groundwater and might not represent the microbial community in a specific location with constant conditions. Since a high-resolution sampling of individual niches was not feasible, the samples represent a broader picture of the alterations in the groundwater microbiome.

#### Changes of α- and β-Diversity in D04, D06, and D11

**Figure 1:**
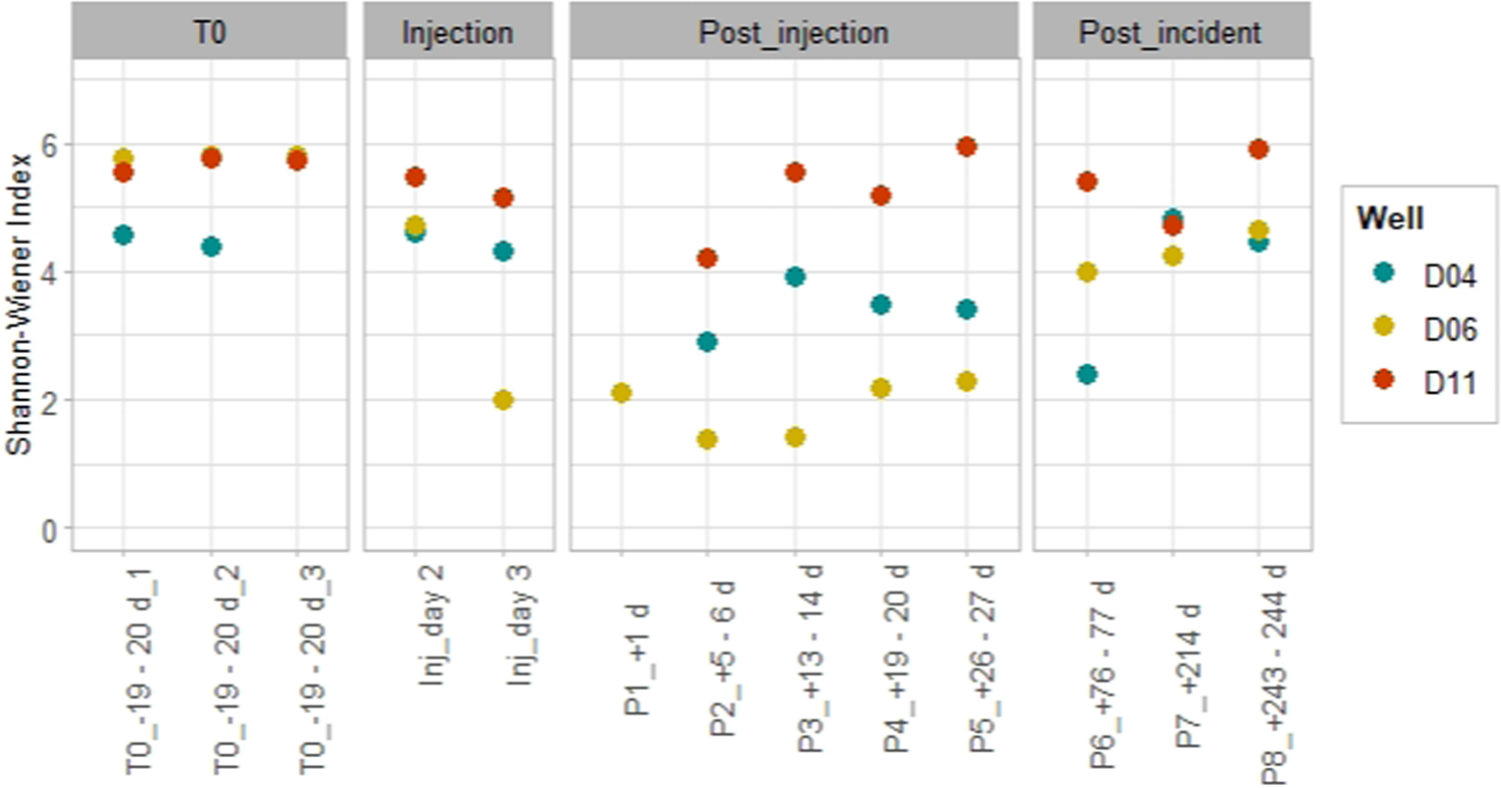
Shannon–Wiener indices showing the α-diversity of the microbial communities in wells D04, D06, and D11 sampled before (T0), during (injection), and after (Post_injection) the H_2_ injection. The H_2_ concentrations in the H_2_-exposed wells fell below the detection limit in the postincident phase (Post_incident).

The Shannon–Wiener indices, representing the α-diversity in the investigated groundwater microbial communities, were between 4.4 and 5.8 (5.4 ± 0.6) in the T0 samples (Figure 1). In well D04, the mean values were the same before (4.5 ± 0.1) and during (4.5 ± 0.2) the H_2_ injection, decreased to 3.4 ± 0.4 post injection, and increased (3.9 ± 1.3) again in the postincident phase, whereby, in this phase, they reached both their minimum (2.4) and their maximum (4.8) 76–77 and 214 days after the H_2_ injection, respectively. The highest Shannon–Wiener index of 5.8 was measured in well D06 before the H_2_ injection, but the mean values decreased over time, from 5.8 ± 0.0 (T0) to 3.4 ± 1.9 (injection) to 1.9 ± 0.4 (Post_injection). The lowest value of 1.4 was measured in well D06 5–6 days post injection. In the postincident phase, the values were higher again on average (4.3 ± 0.3). The data indicate that H_2_ selectively favored the growth of certain prokaryotes capable of consuming H_2_, which then led to a decrease in α-diversity in well D06 (and to a lesser extent in D04) post injection. Supporting this hypothesis, the values were rather stable in the control well D11, ranging between 4.2 and 6.0 (5.4 ± 0.5) (Figure 1), indicating a moderate natural variability in α-diversity as previously observed at the test site.^26^

**Figure 2:**
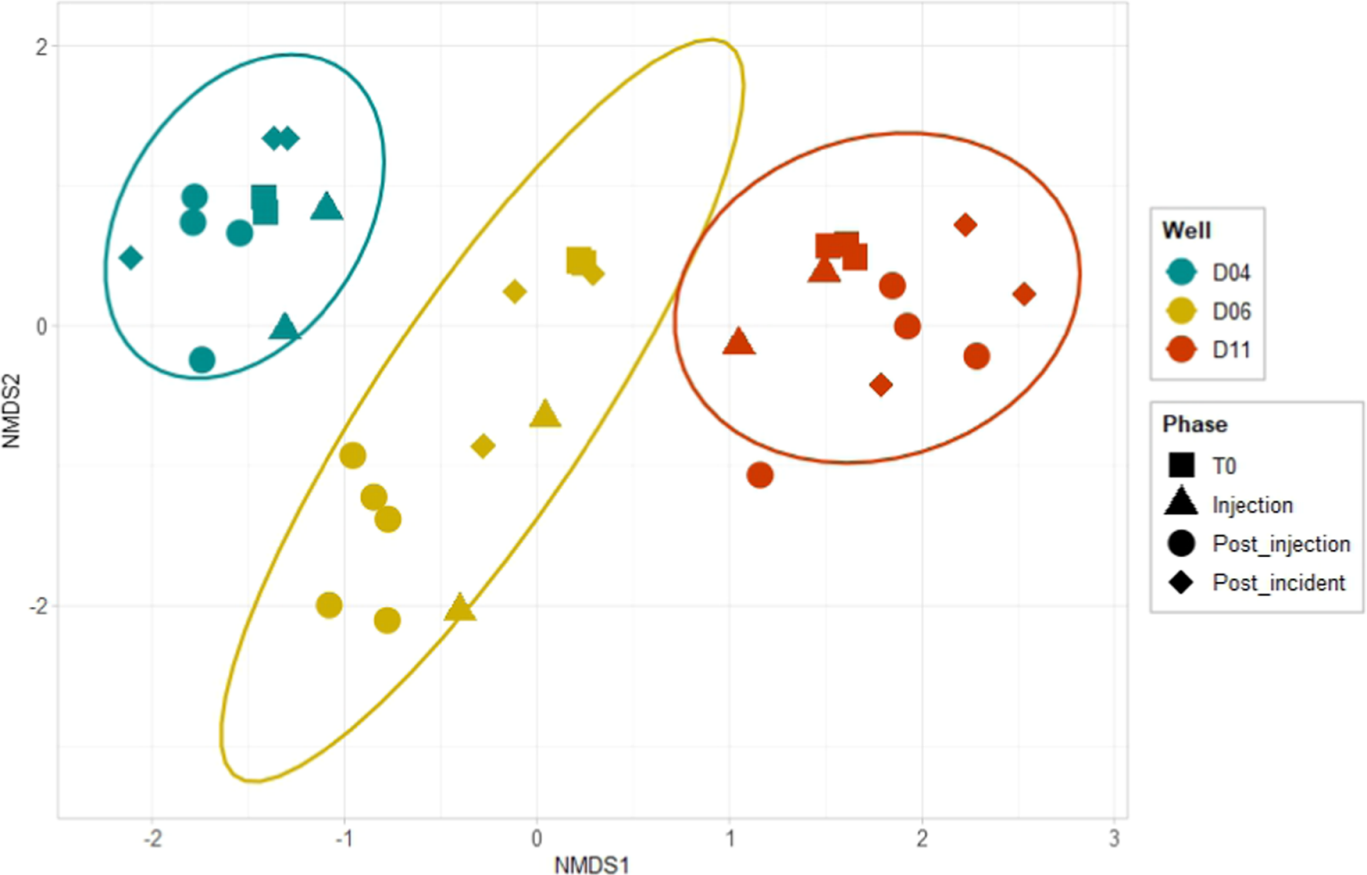
β-diversity illustrated by nonmetric multidimensional scaling (NMDS) using a Bray–Curtis dissimilarity matrix. The microbial communities in the H_2_-exposed wells D04 and D06 and the control well D11 were distinct and, furthermore, microbiome dispersion temporarily changed, especially in well D06 during the injection and postinjection phase.

The NMDS (stress = 0.055) revealed that the samples taken from wells D04, D06, and D11 were distinct, confirming previous results, which showed that the natural groundwater microbiome on the test site is spatially and temporarily heterogeneous.^26^ Notably, the dissimilarities of the microbial communities from the individual wells temporarily increased due to the presence of H_2_, particularly in well D06 (Figure 2). Thus, the presence of H_2_ caused a reduced α-diversity and a changing β-diversity in wells D04 and D06, indicating an enhanced growth of previously low-abundant community members in response to H_2_.

#### Dominant Taxa in Wells D04 and D06 Due to H_2_ Injection, and Putative H_2_ Oxidizers in Well D04

**Figure 3:**
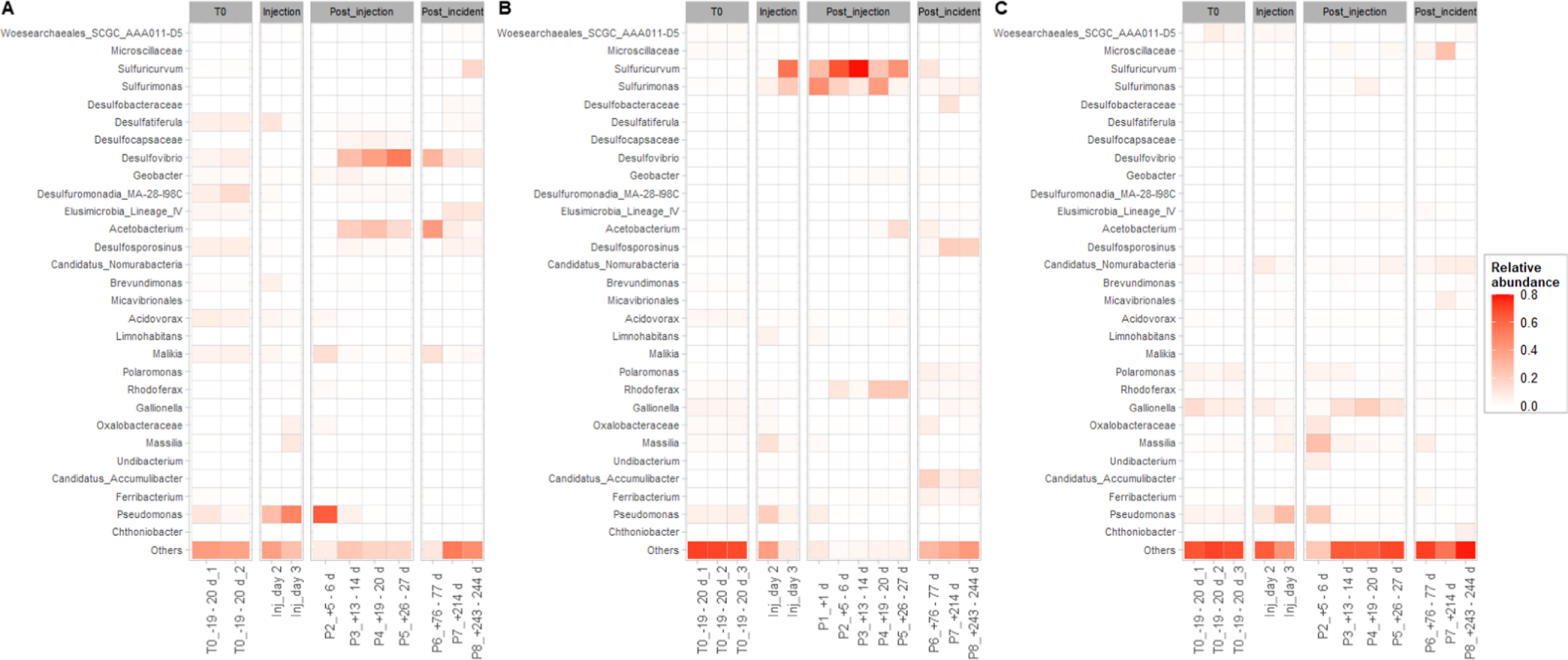
Heatmaps of the most common genera in the H_2_-exposed wells D04 (A) and D06 (B) as well as in the control well D11 (C). Taxa that occurred with >5% relative abundance in at least one sample of the total data set at the genus level were plotted for samples taken before (T0), during (Injection), and after (Post_injection) the H_2_ injection, as well as when the H_2_ concentrations in the H_2_-exposed wells fell below the detection limit (Post_incident).

In well D04, the abundance of phylotypes affiliated to the genus *Pseudomonas* increased during H_2_ injection and early postinjection, making up 63% of the microbial community at most (Figure 3A). *Pseudomonas* species are known to perform various metabolic processes, including H_2_ oxidation.^10,11^ It might be that members of this genus oxidized H_2_ in the initial phase after H_2_ injection, meaning that O_2_ was microbiologically consumed in addition to pure physical stripping by H_2_ injection. However, we cannot rule out any community changes upon the injection phase resulting from displacement of water by the injection process itself; hence, water dominated by *Pseudomonas* phylotypes was transported to well D04. Later, i.e., 13–14, 19–20, 26–27, and 76–77 days after H_2_ injection, *Acetobacterium* and *Desulfovibrio* were the most abundant genera in the groundwater microbial community of well D04 (Figure 3A). Members of both genera are known for the chemoautotrophic or chemoheterotrophic use of H_2_ as an electron donor. Members of the genus *Acetobacterium* are obligately anaerobic and can oxidize H_2_ while reducing CO_2_ via formate to acetate.^40^*Desulfovibrio* species are typical H_2_ oxidizing sulfate reducers, and most strains require, when growing with H_2_ as the energy source, acetate in addition to CO_2_ as a carbon source.^41^ The presence of *Acetobacterium* and *Desulfovibrio* strongly suggests that H_2_ was oxidized anaerobically using carbonate and SO_4_^2–^ as electron acceptors until the beginning of the postincident phase; hydrogenotrophic acetogenesis was also indicated by transiently increasing the concentrations of acetate and formate (Figure S4A,E). Supporting this hypothesis, in microcosm experiments using sediment and groundwater from the test site which were supplied with H_2_, it was shown that homoacetogenesis via H_2_ oxidation and CO_2_ reduction was the dominant H_2_-oxidizing process at elevated H_2_ levels.^18^ The acetate produced by *Acetobacterium* may have promoted the growth of *Desulfovibrio*, as previously observed in H_2_-driven sulfate-reducing bioreactors.^42,43^ Other sulfate-reducing phylotypes popping up post injection belonged to *Desulfosporosinus* and *Desulfocapsaceae* (Figure 3A), also indicating H_2_-driven growth. Notably, the increasing abundances of sulfate-reducing taxa in well D04 in the postinjection phase were not reflected by corresponding decreasing SO_4_^2–^ concentrations; those remained at around 1.3 mM (11.5 m), 1.6 mM (14.5 m), and 1.9 mM (17.5 m) until 246 days after H_2_ injection (Figure S3C), indicating that SO_4_^2–^ concentrations were stratified before H_2_ injection and remained stratified during and after H_2_ injection and that SO_4_^2–^ consumption and SO_4_^2–^ supply were in the steady state in this well. Accordingly, sulfide, the product of dissimilatory sulfate reduction, was detected only sporadically and in low amounts (Figure S3D) and might have precipitated to metal sulfides in the sediment quickly after production; this assumption is supported by decreased concentrations of dissolved iron and manganese species at depths of 14.5 and 17.5 m in the postinjection phase (Figure S6A,B). In the last two groundwater samples collected from well D04 in the postincident period, *Acetobacterium*, *Desulfovibrio*, and *Desulfocapsaceae* phylotypes were less abundant, and the microbial communities became more diverse again (Figure 3A), indicating that specific H_2_ consumers were slowly fading out.

#### Well D06

In well D06, the abundances of *Pseudomonas* phylotypes were slightly increased during the second day of the injection (see the discussion of well D04 above for possible reasons). On the third day of the injection as well as in the postinjection phase, the abundances of phylotypes affiliated with the genera *Sulfuricurvum*, *Sulfurimonas*, and *Rhodoferax* were strikingly increased (Figure 3B). Being facultatively anaerobic and chemolithoautotrophic, members of the genus *Sulfuricurvum* are known for the oxidation of sulfide, sulfite, elemental sulfur (S^0^), thiosulfate, and H_2_ with O_2_ (microaerobic conditions) as well as NO_3_^–^ as electron acceptors.^44,45^*Sulfurimonas* species are widespread and aerotolerant to being facultatively anaerobic. Besides growth on organic compounds, they grow chemolithotrophically with reduced inorganic sulfur compounds or H_2_ as electron donors, NO_3_^–^, NO_2_^–^, or O_2_ as electron acceptors, and CO_2_ or organic compounds like acetate as carbon sources.^46,47^ Hence, the notably high abundances of phylotypes affiliated to *Pseudomonas* and especially to the genera *Sulfuricurvum* and *Sulfurimonas* suggest that H_2_ was oxidized by members of these genera in well D06 during the injection as well as in the postinjection phase, using NO_3_^–^ and O_2_ as electron acceptors. Increasing abundances of *Acetobacterium* in the late postinjection phase indicated anoxic conditions and H_2_ consumption by homoacetogens, similar to well D04, and supported by the detection of acetate and formate (Figure S4E,F). In contrast to well D04, typical sulfate reducers were not abundant in the postinjection phase, indicating that sulfate reduction was not an important electron-accepting process for H_2_ oxidation around well D06. The increased abundances of *Rhodoferax* phylotypes could have been promoted by increased acetate concentrations^48^ provided by homoacetogens; on the other hand, members of the *Rhodoferax* genus may have also oxidized H_2_ using Fe^3+^ as an electron acceptor.^49^ Post incident, *Sulfurimonas* and especially *Sulfuricurvum* became less abundant and the diversity of the microbial communities increased again, with *Desulfosporosinus* and *Candidatus_Accumulibacter* being the most abundant taxa (Figure 3B).

#### Composition of Communities Not Affected by the Presence of H_2_

At the class level, the unaffected microbial communities (samples taken from wells D04 and D06 before H_2_ injection and all samples from the control well D11) were predominantly composed of *Gammaproteobacteria*, *Bacteroidia*, *Parcubacteria*, and *Alphaproteobacteria* (Figure S8) as previously observed at this site.^26^ The detected taxa are considered to be ubiquitous in groundwater habitats, whereby *Gammaproteobacteria* seem to be a common dominant class.^50−53^ At the family or genus level, prior to the injection, the microbial communities in all wells were composed of various taxa, with none of them occurring with an abundance >15%. Phylotypes related to the family MA-28-I98C (belonging to the order *Desulfuromonadales*) and the genera *Pseudomonas* and *Gallionella* were the most abundant taxa.

In control well D11, the compositions of the microbial community were rather stable during the whole investigated time frame, except that phylotypes belonging to *Pseudomonas*, *Massilia*, and *Oxalobacteraceae* were highly abundant in the samples taken on the third injection day as well as 5–6 days post injection. Notably, these genera also showed increased abundances in well D06 on the second day of the injection, as well as in well D04 on the third day of the injection. While microbial community members potentially capable of H_2_ oxidation quickly became dominant in the H_2_-exposed wells (see the discussion above), the abundances of *Pseudomonas*, *Massilia*, and *Oxalobacteraceae* seem to continually increase in well D11 from the second day of the injection to 5–6 days after the H_2_ injection. The sample collected 5–6 days post injection additionally showed the lowest value in α-diversity and the highest dispersion in β-diversity in well D11 (Figures 1 and 2). This indicated that the gas injection with the concomitant increase in the pressure head leads to a displacement of the surrounding waterbody, hypothesized already by the geochemical data.^18^ Nevertheless, the H_2_ concentrations were below the detection limit in well D11 during the entire monitoring period. Besides probably being influenced by the pressure effect of the injection and the concomitant waterbody displacement, the sampled communities from control well D11 showed a moderate level of natural variability within the nine months of monitoring, which corresponds to previous studies focusing on the spatial and temporal variabilities of aquifer microbial communities.^26,54−58^

### Total Cell Count Changes Due to H_2_ Injection

**Figure 4:**
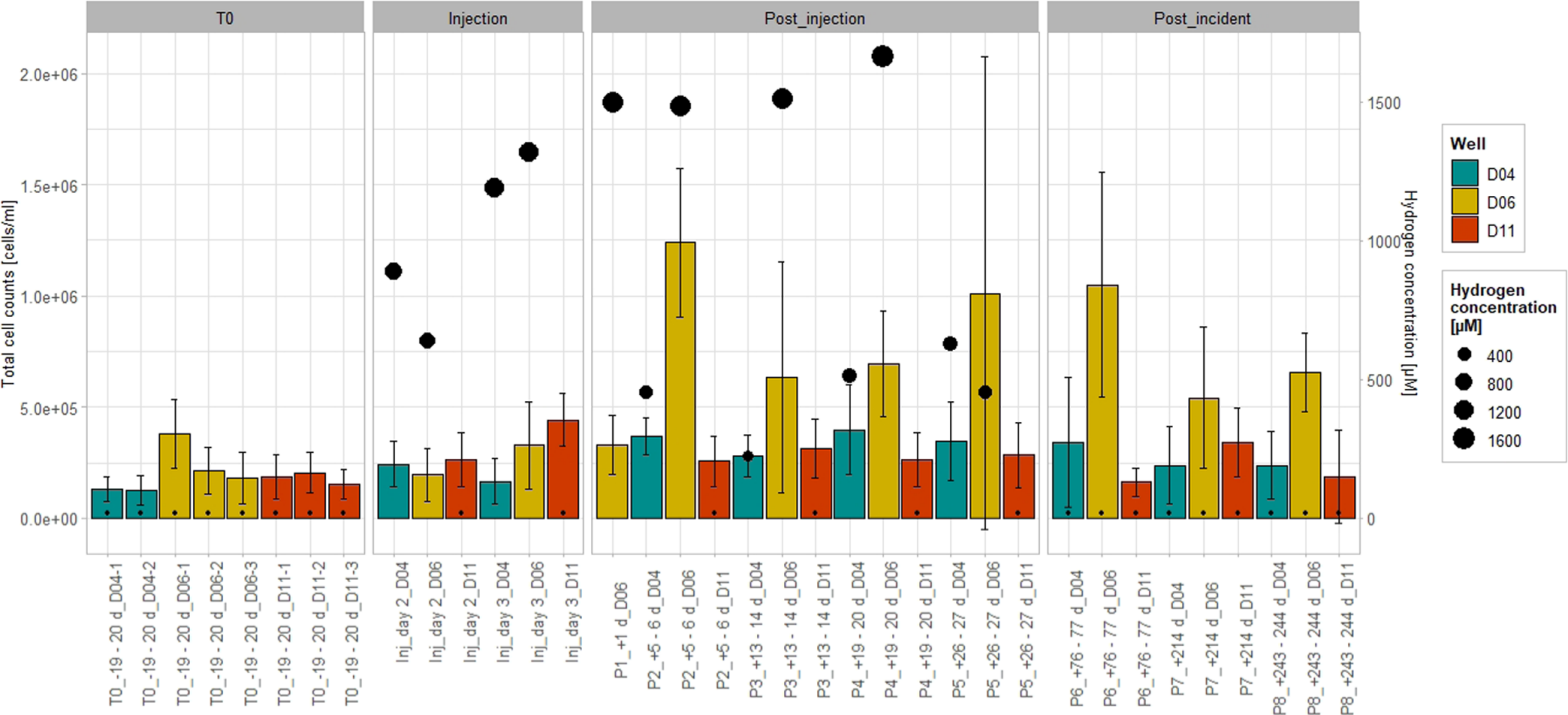
Total cell counts of samples from the H_2_-exposed wells D04 and D06 as well as the control well D11 taken before (T0), during (Injection), and after (Post_injection) the H_2_ injection, as well as when the H_2_ concentrations in the H_2_-exposed wells fell below the detection limit (Post_incident). The H_2_ concentrations are also shown (black dots).

The total cell counts in samples taken before the H_2_ injection were between 1.3 × 10^5^ and 3.8 × 10^5^ cells/mL, which is within the range determined at natural conditions (3.2 × 10^4^–2.0 × 10^6^ cells/mL) in our previous study^26^ and, furthermore, within the range that would be expected for pristine groundwater, i.e., 10^4^–10^6^ cells/mL.^59^ During and after the H_2_ injection, the values determined in samples from the H_2_-exposed well D04 and the control well D11 did not rise above 4 × 10^5^ cells/mL, but those from the H_2_-exposed well D06 increased up to (1.2 ± 0.3) × 10^6^ cell/mL 5–6 days post injection (Figure 4). This number is still within the normal range of the 10^4^–10^6^ cells/mL expected in pristine groundwater. The elevated total cell counts in well D06 detected after H_2_ injection could be due to the stimulated growth of *Sulfuricurvum* and *Sulfurimonas*. H_2_ oxidation using O_2_ or NO_3_^–^ as the electron acceptor is energetically more favorable than sulfate or carbonate-dependent H_2_ oxidation,^8^ which could explain why significantly increased cell counts were measured in well D06 but not in well D04, where microbial community data suggest predominantly anaerobic H_2_ oxidation by *Acetobacterium* and *Desulfovibrio*.

### Environmental Implication of Gaseous H_2_ Injection

In summary, the data demonstrate that the injected H_2_ was rapidly oxidized by various indigenous, ecophysiologically different microorganisms, leading to decreased α-diversity and concurrently changed β-diversity. In well D04, increased abundances of sulfate-reducing and acetogenic taxa indicated H_2_ consumption primarily by the use of SO_4_^2–^ and CO_2_ as electron acceptors, respectively. In well D06, high abundances of aerobic, nitrate-reducing, iron-reducing, and (later) acetogenic taxa indicated H_2_ oxidation primarily by the use of O_2_, NO_3_^–^, Fe^3+^, and later CO_2_ as electron acceptors. The presented data confirm the results of previously published δ^2^H analyses of H_2_, which indicated microbial consumption of H_2_ in wells D04 and D06.^18^ The assumed consumption of H_2_ by microbes using different electron acceptors (O_2_, NO_3_^–^, Fe^3+^, SO_4_^2–^, or CO_2_) indicated by the microbial community analyses was not concomitant with a corresponding clear in situ depletion of all of these electron acceptors in the investigated wells, although the aquifer was locally saturated with H_2_ during injection. Hence, oxidation of H_2_, reduction of electron acceptors, and subsequent supply of electron acceptors by groundwater flow may have been in a steady state; furthermore, end products of electron acceptor reduction were likely transformed (acetate) or precipitated (FeS). Considerable amounts of H_2_ might have been distributed in the aquifer by physicochemical processes, perhaps resulting in outgassing of an unknown amount of the injected H_2_. Notably, the H_2_-induced effects on the microbial community were shown to be temporarily limited as α- and β-diversity approached the initial state again at the end of the monitoring period, resulting in no persistent negative effects on the groundwater microbiome in the course of the investigated underground H_2_ leakage scenario. On the other hand, in the case of a continuous H_2_ leak from an underground storage site, spatially separated microbial communities likely develop alongside the H_2_ plume depending on the availability of electron acceptors, a scenario that is well-known in aquifers contaminated by aromatic hydrocarbons degradable with various electron acceptors.^60^

## Supporting Information Available

Additional details on materials and methods (SI-1) including field site evaluation (SI-1.1), injection of H_2_ (SI-1–2, Figure S1), groundwater sampling (SI-1.3), and statistical analyses (SI-1.4); additional results (SI-2) on the geochemical conditions in the sampled wells (SI-2.1, Figures S2–S6) and on 16S rRNA gene sequencing (SI-2.2, Figures S7 and S8); references for SI-1 and SI-2 (PDF)
