# Supplementary material for: Rapid Consumption of Dihydrogen Injected into a Shallow Aquifer by Ecophysiologically Different Microbes
Source: Environ Sci Technol. 2023 Dec 20;58(1):333–41. doi: 10.1021/acs.est.3c04340 (PMC10785757; doi:10.1021/acs.est.3c04340)
Supplement: Supplementary file 1 — es3c04340_si_001.pdf [file es3c04340_si_001.pdf]

Supporting Information

Rapid consumption of dihydrogen injected into a shallow aquifer by ecophysiologically different microbes

Nina S. Keller[a], Klas Lüders[b], Götz Hornbruch[b], Susann Birnstengel[c], Carsten Vogt[a]*, Markus Ebert[b], René Kallies[d], Andreas Dahmke[b], & Hans H. Richnow[a,e]

[a] Helmholtz Centre for Environmental Research – UFZ, Department Isotope Biogeochemistry, 04318 Leipzig, Germany

[b] University of Kiel, Institute for Geosciences, 24118 Kiel, Germany

[c] Helmholtz Centre for Environmental Research – UFZ, Department Monitoring & Exploration Technologies, 04318 Leipzig, Germany

[d] Helmholtz Centre for Environmental Research – UFZ, Department Environmental Microbiology, 04318 Leipzig, Germany

[e] Isodetect GmbH, Deutscher Platz 5b, 04103 Leipzig, Germany (current address).

Number of pages: 16

Number of figures: 8

Number of tables: 0

S. 2

SI-1 Materials & Methods

SI-1.1 Evaluation of hydrogeological conditions

The hydrogeological properties of the aquifer at the field site were evaluated by the hydraulic profiling tool (HTP, Geoprobe®, USA) [1, 2]. The hydraulic gradient showed values around 5 ‰ in the area of the dihydrogen (H_2) injection (see SI-1.2), determined in March 2018 and October 2020. Saturated hydraulic water conductivities were around 1.5 E^{-4} m s^{-1}, determined by sediment analyses in a cylindrical core-cutter and sieve analyses. The groundwater flow velocity was calculated to be around 0.3 m d^{-1}, assuming an effective porosity of 25% [2].

SI-1.2 Injection of H_2

Gaseous H_2 (N5.0; AirLiquide, France), installed in two racks of cylinders, was injected in a depth of about 18 m below ground level (bgl) by means of three continuous multichannel tubes (3-CMT; Solinst©, Canada) installed using SonicSampDrill system transversally to the assumed flow direction in a distance of about 2 m [2]. The gas cylinders were connected to three explosion protected mass flow controllers (red-y Typ GIC-C4SU-BB26; Vögtlin Instruments GmbH, Muttenz, Switzerland) and further to the three injection wells by suitable gas tubes. The mass flow was regulated and logged with the software Easy-HTK (HTK – High Tech Kommunikationssysteme GmbH & Co. KG, Ellerstadt, Germany). Around 12 kg of H_2 were injected intermittently in seven injection phases (1 x 1h, 1 x 2h, 1 x 3h, and 4 x 6h) and six interruptions (1 x 1h, 1 x 2h, 1 x 3h, and 3 x 6h); hence the injection lasted 54 hours (Figure S1 C). The H_2 was injected through a 20 cm filter screen between 18.1 and 18.3 m bgl with a slight overpressure (~ 0.3 MPa) considering a water saturated zone of 15 m above the injection

depth, aiming to generate a coherent gas phase and dissolved H_2-plume. The filter screen of the injection lances was overlayed by bentonite bands to minimize gas migration upwards along the tube. For the first three hours, the flow rate was 13.4 mol min^{-1} (300 NL/min) H_2. Due to outgassing in monitoring wells close to the injection well, the flow rate was then reduced to 1.8 mol min^{-1} H_2 (40 NL/min) for the next 27 hours. Potential gas leakages at connections in the surface installations, at the injection wells and at surrounding monitoring wells were frequently monitored with multi-gas detectors. Furthermore, the whole injection zone was externally ventilated to inhibit the formation of explosive atmospheres. More details of the injection are given elsewhere [2].

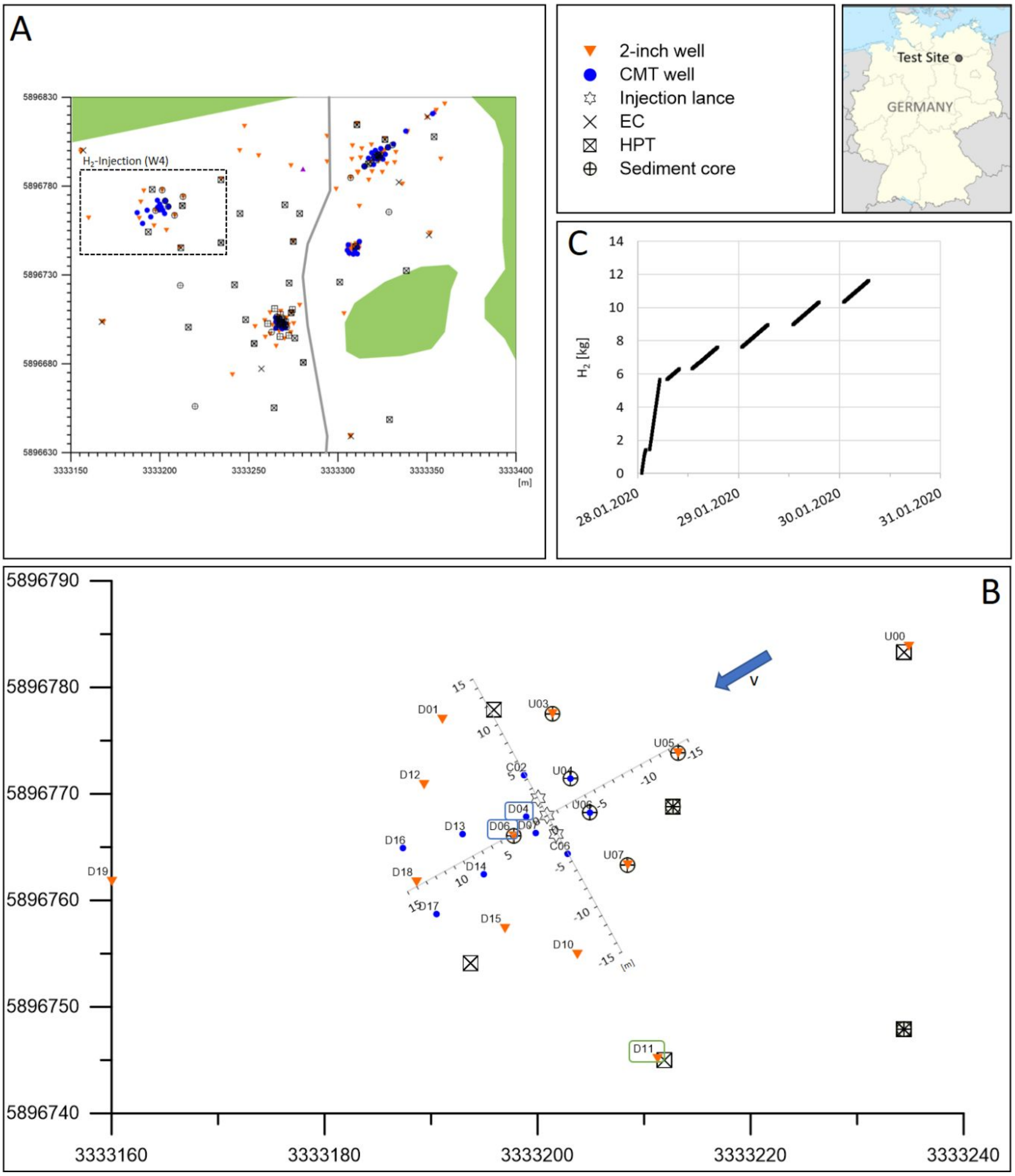

Figure S1. Location of the hydrogeological TestUM test site near Wittstock/Dosse in Germany (top, right corner), area of the H_2 injection experiment at the test site (A), location of the monitoring wells at the H_2 injection area, highlighting the investigated H_2-influenced wells D04 and D06 close to the injection wells and the reference well D11 (B), and mode of H_2 injection (C). CMT = continuous multichannel tubing; EC = electrical conductivity logs; HTP = hydraulic profiling tool.

SI-1.3 Groundwater sampling

Groundwater was sampled as described elsewhere [2]. Wells D06 and D11 are 2 in. wells (UTK-EcoSense GmbH, Germany), and well D04 is a CMT well (Solinst, Canada). Wells were lined with high-density polyethylene (HDPE). Groundwater was pumped up from the CMT well (D04) with a peristaltic pump (24V DC 125 rpm, Verder Deutschland GmbH & Co. KG, Germany) and from the 2 in. wells (D06, D11) with a submersible pump (Whale submersible electric galley pump, Munster Simms Engineering, Ltd., Northern Ireland). Subsequently, pH, electrical conductivity, oxygen concentration, and redox potential of the groundwater were determined on site by an array of multiparameter probes (ProfiLine 1970i, WTW, Germany). For concentration analyses of dissolved H_2, 20 mL gas chromatography (GC) vials (pre-filled with 1.8 g of NaCl to inhibit microbial H_2 conversion) were filled with 5 mL of water and immediately closed with gas-tight butyl stoppers and aluminium crimp caps. The vials were stored upside down in a refrigerator until analysis. For sulfide determinations, 5 mL groundwater samples were filtered (0.2 µm) *on site* and stored cool in 8 mL disposable centrifuge tubes (Sarstedt AG & Co. KG, Germany) after stabilization with three drops of 1 M NaOH. Groundwater samples for analyses of anions, cations, CH_4, TCE, acetate and formate were filtered (0.2 µm) *on site*, filled in glass vials without headspace, closed gas-tight and stored at 4°C until analysis.

SI-1.4 Amendment: Figures & statistical analyses

Figures and statistical analyses were conducted with RStudio version 2022.02.1.+461 [3]. To show the changes of the most common microorganisms up to the class and genus level in the microbial communities from wells D04, D06, and D11 over time, the relative abundances of

amplicon sequencing variants (ASVs) that occurred with > 2% in at least one sample were plotted in heatmaps and barplots using the function *ggplot()* (*ggplot2* [4] and *scales* [5] packages). In advance, the respective data frame had been converted into a single column format with the *melt()* function (*reshape2* package [6], and a colour assignment had been done with the *RColorBrewer* package [7].

The Quantitative Insights Into Microbial Ecology 2 (QIIME 2) biom and qza files were imported and merged using *phyloseq* [8] and *qiime2R* [9] packages. With the function *rarecurve()* (*vegan* [10] package), rarefaction curves were drawn of the unrarefied data to assess the sampling depth (Figure S7 [A]). The rarefaction was done with the function *rarefy_even_depth()* (*phyloseq* package). The sampling depth was set at 37,219, i. e., the minimum number of reads gathered, since all curves had reached plateau and no sample had to be excluded. Curves of the unrarefied and rarefied data were plotted with the function *ggrare()* (https://github.com/gauravsk/ranacapa/blob/master/R/ggrare.R) as well as functions from the *ggplot2* package and the function *ggarrange()* (*ggpubr* [11] package).

As a proxy of the alpha diversity, the Shannon-Wiener indices of the rarefied data were plotted with the function *plot_richness()* (*phyloseq* and *ggplot2* packages). The beta diversity of the rarefied data was illustrated performing non-metric multidimensional scaling (NMDS), i. e., an unconstrained distance-based ordination that shows similarities/dissimilarities between samples taken from wells D04, D06, and D11 and, respectively, between samples collected during the different phases. The function *ordinate()* (*phyloseq* package; *bray* distance, k = 3) was used to conduct the NMDS. A Bray-Curtis dissimilarity matrix was chosen since it is not affected by zero values between samples. Wisconsin double standardization and square root transformation, which help to mitigate the stress, the weight of the dominant ASVs, as well as the double-zero problem, were automatically done. The number of dimensions was adjusted

to k = 3 in order to further reduce the stress. The figure was created with the function *plot_ordination()* (*phyloseq* and *ggplot2* packages).

The total cell counts were plotted together with the H_2 concentrations using the function *ggplot()* (*ggplot2* package).

SI-2 Results

SI-2.1 Geochemical conditions in wells D04, D06, and D11

For each well (D04, D06, and D11), data for three different depths (11.5 m, 14.5 m, and 17.5 m) and for a time period of up to 78 days before H_2 injection and up to 316 days after H_2 injection are given. Generally, data for 11.5 m depth are presented in yellow circles, data for 14.5 m depth in green diamonds, and data for 17.5 m depth in blue triangles. The time of H_2 injection is defined as day 0 and indicated by a dashed line.

In Figure S2 A-L, time courses of H_2 concentrations (A: D04; E: D06; I: D11), redox potential (B: D04; F: D06; J: D11), alkalinity (C: D04; G: D06; K: D11), and pH (D: D04; H: D06; L: D11) are shown. In Figure S3 A-L, time courses of NO_3^- (A: D04; E: D06; I: D11), NO_2^- (B: D04; F: D06; J: D11), SO_4^{2-} (C: D04; G: D06; K: D11), and sulfide (D: D04; H: D06; L: D11) concentrations are presented. In Figure S4 A-L, time courses of acetate (A: D04; E: D06; I: D11), formate (B: D04; F: D06; J: D11), methane (C: D04; G: D06; K: D11), and trichlorethylene (TCE) (D: D04; H: D06; L: D11) concentrations are shown. In Figure S5 A-L, time courses of Na^+ (A: D04; E: D06; I: D11), Cl^- (D: D04, F: D06; J: D11), Ca^{2+} (C: D04; G: D06; K: D11), and Mg^{2+} (D: D04; H: D06; L: D11) concentrations are shown. In Figure S6 A-F, time courses of dissolved iron (A: D04; C: D06 E: D11) and dissolved manganese species (B: D04; D: D06; F: D11) concentrations are shown.

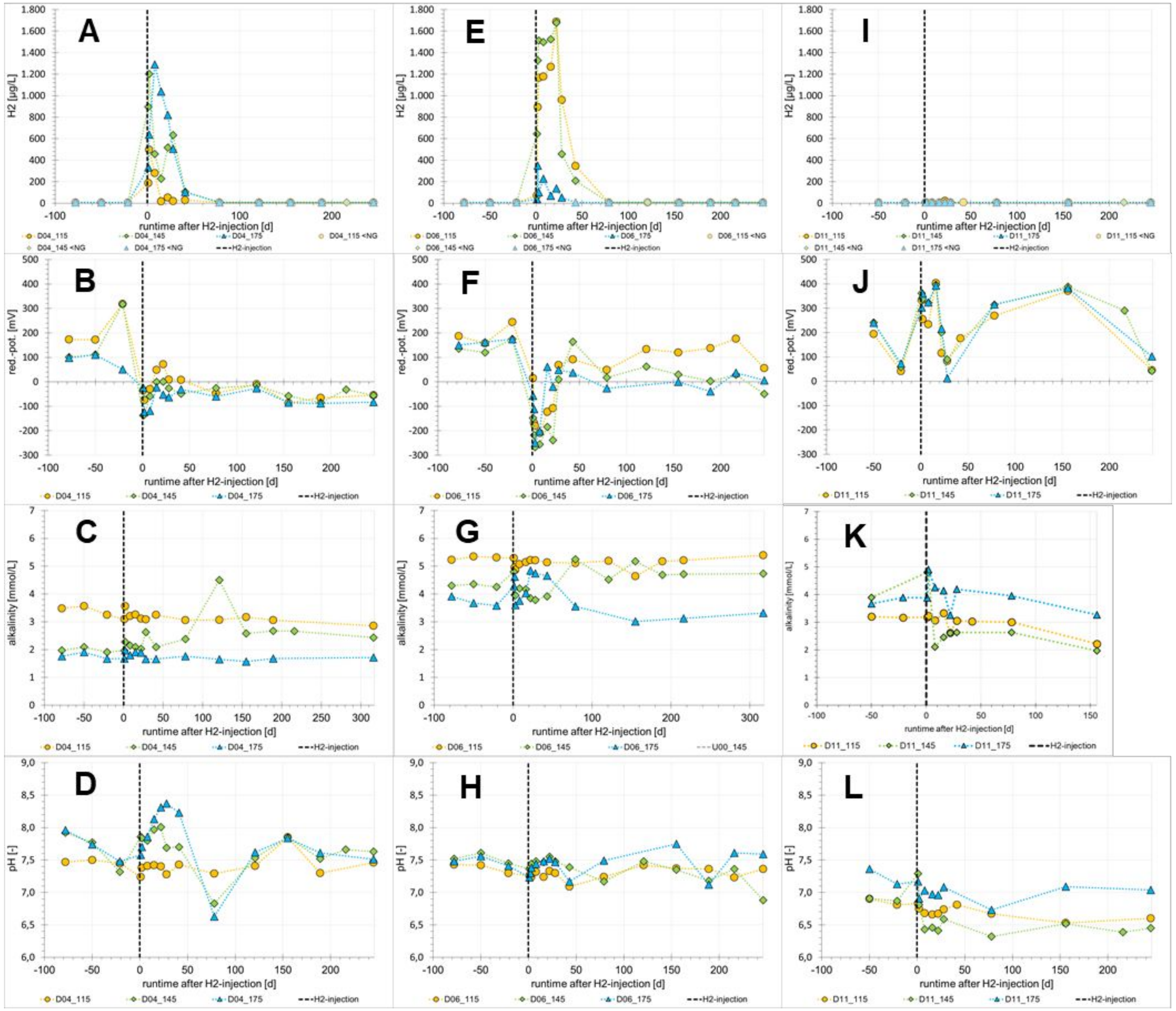

Figure S2. Time courses of H_2 concentrations (A: D04; E: D06; I: D11), redox potential (B: D04; F: D06; J: D11), alkalinity (C: D04; G: D06; K: D11), and pH (D: D04; H: D06; L: D11).

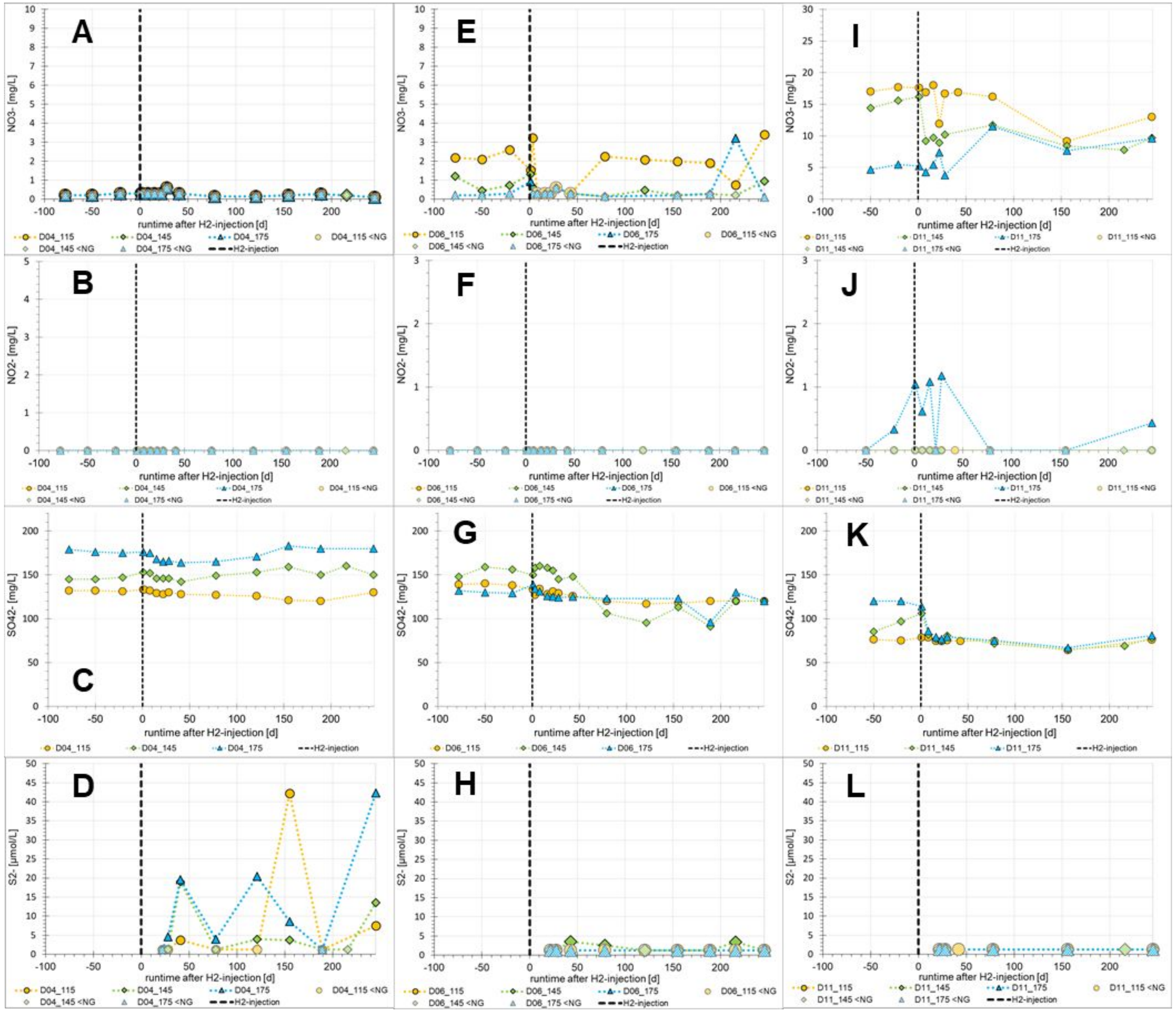

Figure S3. Time courses of concentrations of NO_3^- (A: D04; E: D06; I: D11), NO_2^- (B: D04; F: D06; J: D11), SO_4^{2-} (C: D04; G: D06; K: D11), and sulfide (D: D04; H: D06; L: D11).

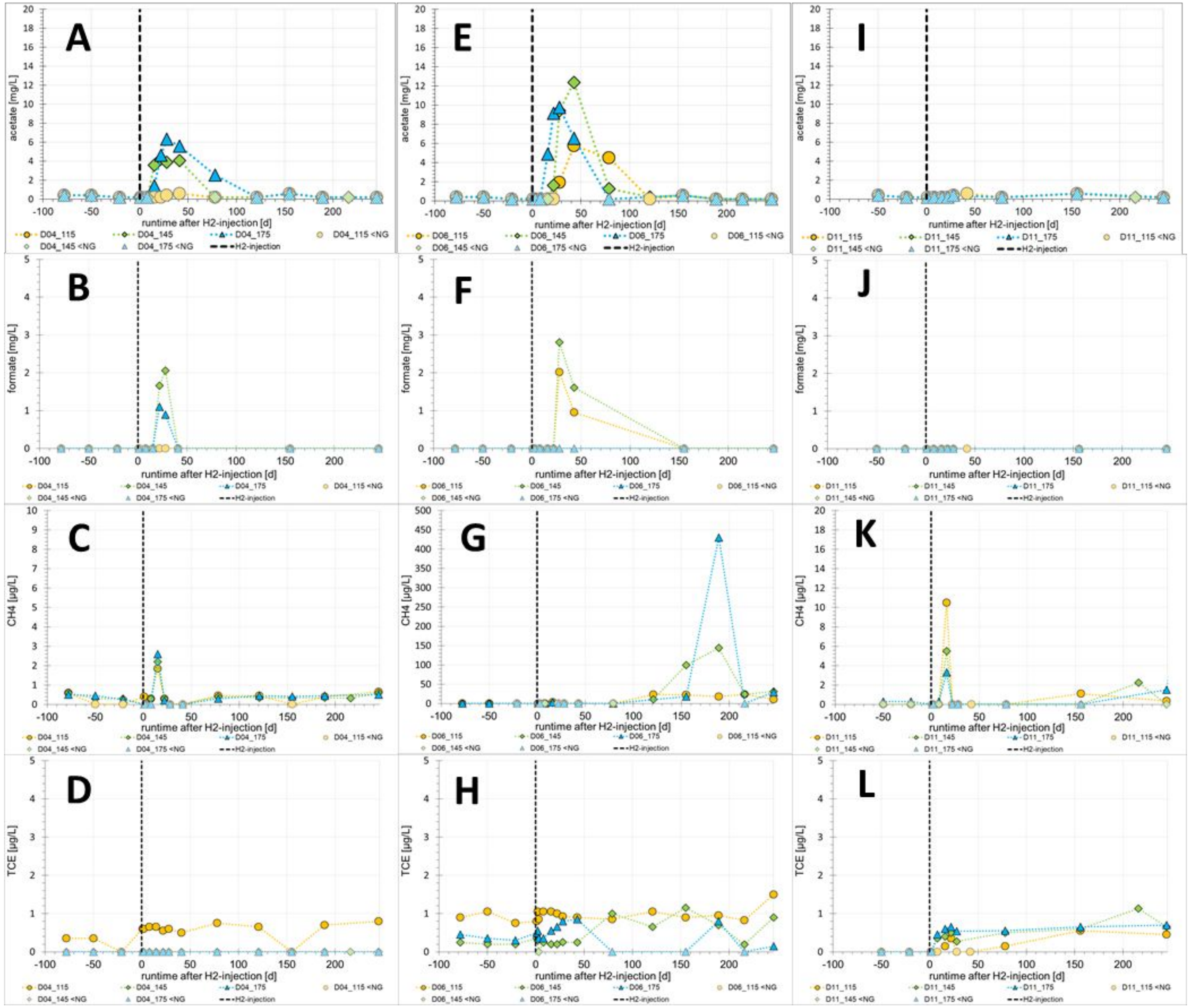

135

136 **Figure S4.** Time courses of concentrations of acetate (A: D04; E: D06; I: D11), formate (B: D04; F: D06; J: D11), CH_4 (C: D04; G: D06; K: D11), and TCE (D: D04; H: D06; L: D11).

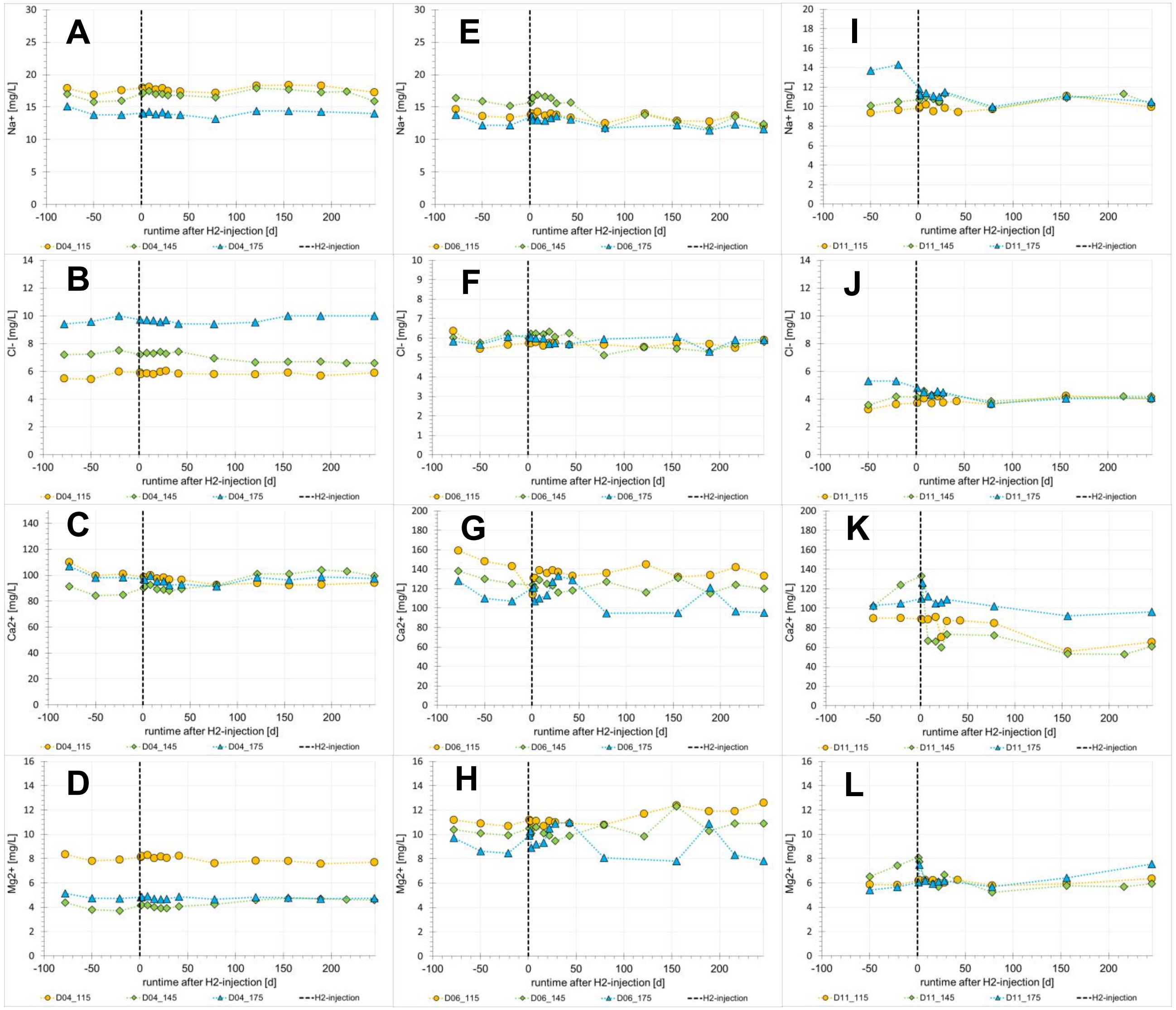

Figure S5. Time courses of concentrations of Na^+ (A: D04; E: D06; I: D11), Cl^- (B: D04; F: D06; J: D11), Ca^{2+} (C: D04; G: D06; K: D11), and Mg^{2+} (D: D04; H: D06; L: D11).

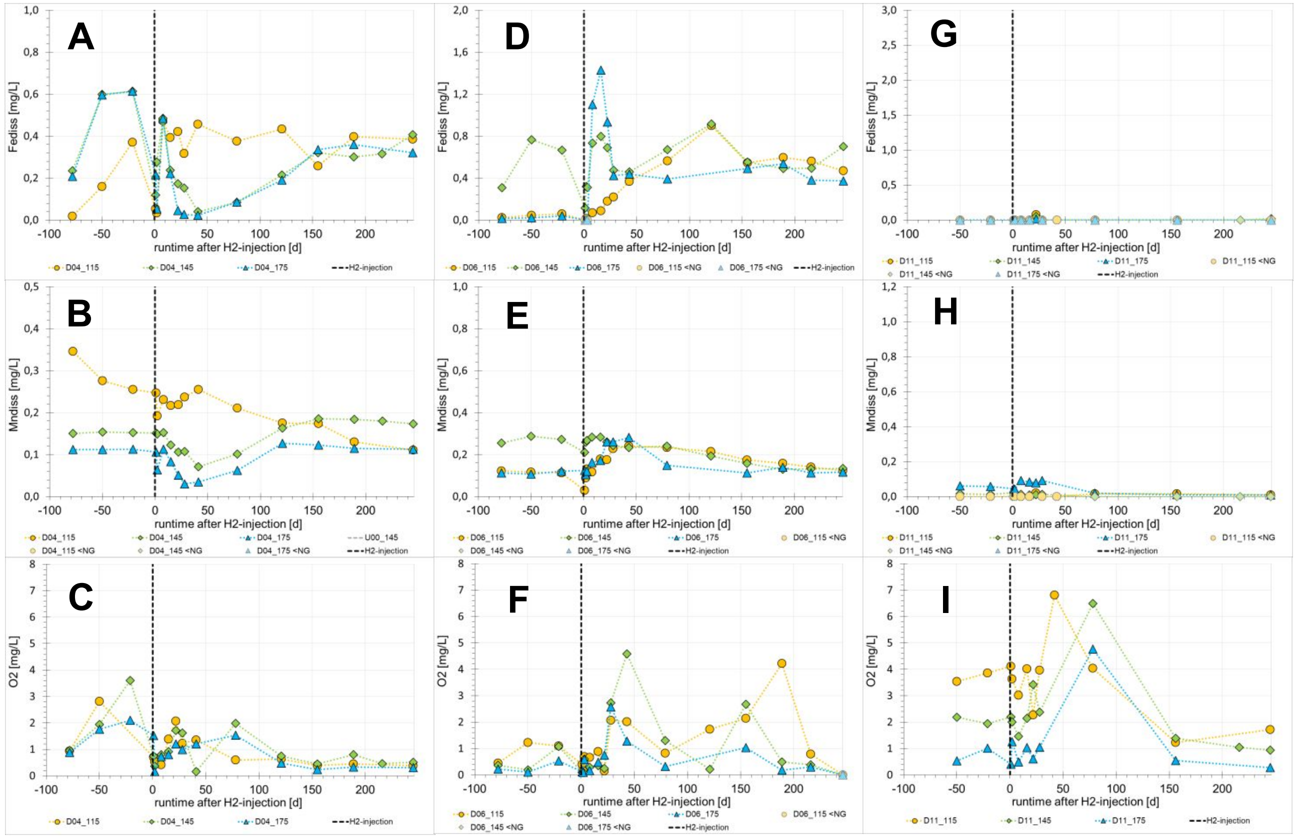

Figure S6. Time courses of concentrations of dissolved iron species (A: D04; D: D06; G: D11), dissolved manganese species (B: D04; E: D06; H: D11), and dissolved oxygen (C: D04; F: D06; I: D11).

SI-2.2 *16S rRNA* gene amplicon sequencing

In the *16S rRNA* gene amplicon sequence analysis, a total of 2,366,867 reads were gathered, ranging from 37,219 to 90,686 reads per sample (65,746.31 ± 14,147.34), and a total of 7431 ASVs were identified. Due to rarefaction with a sampling depth set to the minimum number of reads, 532 ASVs were removed. Nevertheless, curves of the rarefied data all plateaued, showing that samples were sequenced deeply enough to capture full community diversity (Figure S7).

In Figure S8, the most abundant classes in the H_2-exposed wells D04 (A) and D06 (B) as well as in reference well D11 (C) are shown.

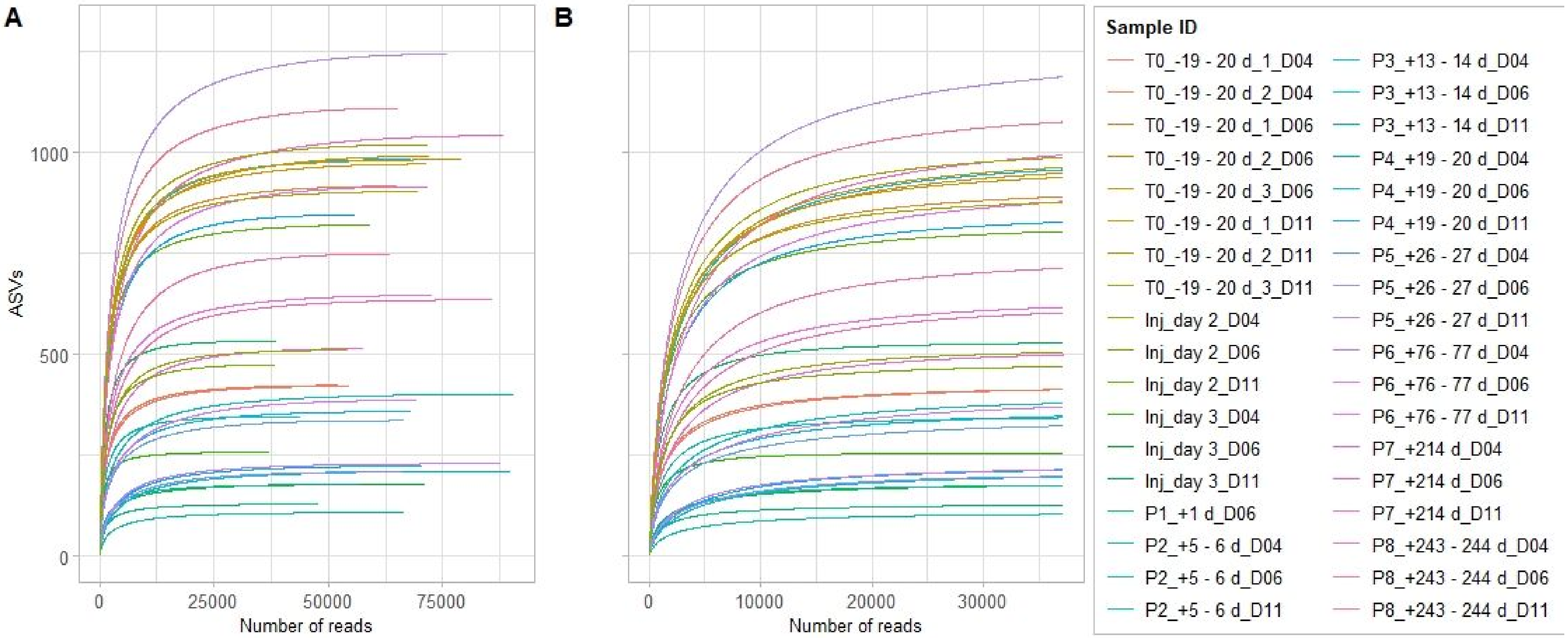

Figure S7. Curves of the unrarefied (A) and rarefied (B) dataset. Between 37,219 and 90,686 reads per sample were gathered (65,746.31 ± 14,147.34, giving a total of 2,366,867 reads) and 7431 ASVs were classified, which belonged to bacteria and archaea. The sampling depth for rarefaction was set to the minimum number of reads; due to rarefaction, 532 ASVs were removed from the dataset. The rarefaction curves all plateaued, showing that samples were sequenced deeply enough to capture the full diversity of the microbial communities.

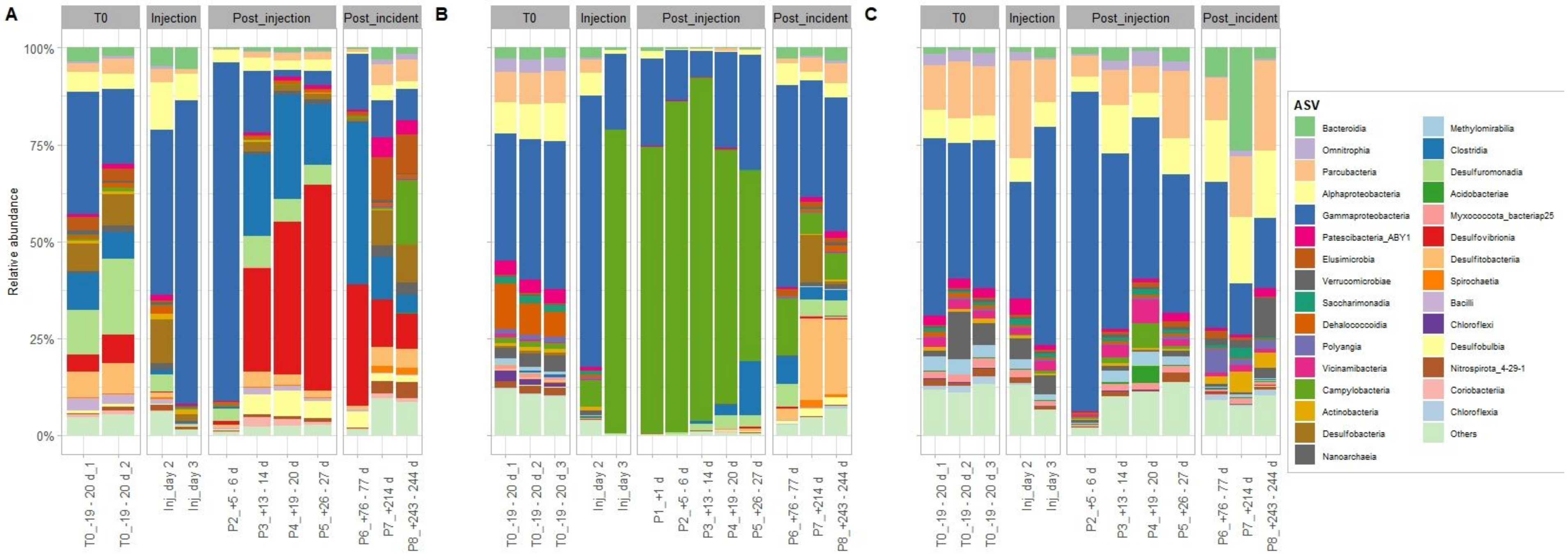

Figure S8. Barplots of the most abundant classes in the H_2-exposed wells D04 (A) and D06 (B) as well as in reference well D11 (C). Taxa that occurred with > 2% relative abundance in at least one sample of the total data set at the class level were plotted faceting samples taken before (T0), during (Injection), and after (Post_injection) the H_2 injection, as well as when the H_2 concentrations in the H_2-exposed wells fell below the detection limit (Post_incident). The unaffected microbial communities (i. e., T0 samples and samples from reference well D11) were dominated by *Gammaproteobacteria*, *Parcubacteria*, *Alphaproteobacteria*, and *Bacteroidia*. In well D04, *Gammaproteobacteria* was still the most abundant class during the injection as well as 5 – 6 days post injection, whereas, *Desulfovibrionia* and *Clostridia* occurred with the highest abundances 13 – 14, 19 – 20, 26 – 27, and 76 – 77 days after the H_2 injection. In well D06, the abundance of *Gammaproteobacteria* was increased on the second day of the injection. However, on the third day of the injection as well as in the post injection phase, *Campylobacteria* was the most abundant class.

References

1. McCall, W. and T.M. Christy, *The Hydraulic Profiling Tool for Hydrogeologic Investigation of Unconsolidated Formations.* Ground Water Monitoring and Remediation, 2020. **40**(3): p. 89-103.
2. Loffler, M., et al., *Stable Hydrogen Isotope Fractionation of Hydrogen in a Field Injection Experiment: Simulation of a Gaseous H-2 Leakage.* Acs Earth and Space Chemistry, 2022. **6**(3): p. 631-641.
3. RStudio Team, *RStudio: Integrated Development Environment for R*. 2022, RStudio, PBC: Boston, MA.
4. Wickham, H., *Package ‘ggplot2’: elegant graphics for data analysis.* Springer-Verlag New York. doi, 2016. **10**: p. 978-0.
5. Wickham, H., et al., *scales: Scale Functions for Visualization.* 2022.
6. Wickham, H., *Reshaping data with the reshape package.* Journal of Statistical Software, 2007. **21**(12): p. 1-20.
7. Neuwirth, E., *RColorBrewer: ColorBrewer Palettes.* 2022.
8. McMurdie, P.J. and S. Holmes, *phyloseq: an R package for reproducible interactive analysis and graphics of microbiome census data.* PLoS One, 2013. **8**(4): p. e61217.
9. Bisanz, J.E., *qiime2R: Importing QIIME2 artifacts and associated data into R sessions.* 2018.
10. Oksanen, J., et al., *vegan: Community Ecology Package.* 2022.
11. Kassambara, A., *ggpubr: 'ggplot2' Based Publication Ready Plot.* 2020.
